# Benefit finding among family caregivers of patients with advanced cancer in a palliative treatment: a qualitative study

**DOI:** 10.1186/s12912-024-02055-z

**Published:** 2024-06-11

**Authors:** Yuanyi Song, Min Wang, Meina Zhu, Na Wang, Ting He, Xu Wu, Zhihui Shi, Mengye Chen, Tian Ji, Ying Shen

**Affiliations:** 1 Department of Breast and Urological Oncology, Department of Radiation and Medical Oncology, Zhongnan Hospital of Wuhan University, Wuhan University, Wuhan, Hubei China; 2 Department of Thyroid and Breast Surgery, Zhongnan Hospital of Wuhan University, Wuhan University, Wuhan, Hubei China

**Keywords:** Benefit finding, Advanced cancer, Palliative, Caregivers, Care, Positive, Qualitative

## Abstract

**Background:**

Benefit finding is the search for positive meaning from traumatic events, such as cancer. It can help caregivers have a positive experience in the caregiving process, relieve negative emotions, and reduce caregiving stress. The aim of this study was to explore benefit finding among caregivers of patients with advanced cancer in their palliative caregiving journey.

**Methods:**

An exploratory qualitative design of phenomenology was used. Semistructured interviews were conducted with 19 caregivers of palliative care patients with advanced cancer. The Colaizzi 7-step analysis was used to analyse, summarize, and extract themes from the interview data.

**Results:**

The study identified five themes of caregiver benefit finding in the caregiving process: personal growth, strengthened relationships with patients, adjustment and adaptation, perceived social support, and perceived meaning in life. Most caregivers reported a closer, more dependent relationship with the patient, and only one caregiver did not report any positive changes.

**Conclusions:**

Caregivers of palliative care patients with advanced cancer can have positive experiences in their care. Healthcare professionals should focus on supporting caregivers and helping them find positive experiences to cope with the challenges of caregiving and improve their quality of life.

## Background

According to 2020 data, there were 19.29 million new cancer cases and 9.96 million cancer-related deaths worldwide, and the incidence and mortality of cancer are increasing rapidly [1]. With advances in cancer treatment, palliative care provides comprehensive care for patients with advanced cancer, aiming to control symptoms such as pain, improve the quality of life for patients and family caregivers, preserve dignity and comfort, and positively affect the disease process as much as possible [2]. Benefit finding refers to seeking positive meaning from a traumatic event such as cancer [3]. It is the identification of benefits in the face of adversity and plays an important role in the cognitive process of adapting to adversity, a positive change that involves aspects of spiritual growth and a healthy lifestyle, an adaptive response to an adversarial situation [4].

Informal caregivers (spouses/partners, family members, close friends, etc.) play a crucial role in providing daily care and support to individuals with advanced cancer [5, 6]. To meet the palliative care needs of people with advanced cancer, physical, psychological, spiritual, communication, decision-making, and financial issues may be addressed [7]. Caregiving is a complex and ever-evolving task. As the disease progresses, the caregiving burden gradually increases, often resulting in anxiety, depression, and fatigue among caregivers [8]. Nonetheless, positive psychology shows that caregivers can also experience positive changes, such as personal growth and closer relationships with others [9–11]. These positive meanings have the potential to enhance patient-caregiver interactions [9], redefine the meaning of life, and strengthen interpersonal relationships and self-perception [12].

The physical and mental health of patients and caregivers are interdependent and mutually influential [13]. Caregivers adapt to the stress caused by cancer by evaluating and adjusting coping mechanisms [14, 15]. Benefit finding can assist caregivers in actively adapting to their role [16] and alleviating negative emotions and psychological distress [17]. Some studies have demonstrated that caregivers experience positive changes associated with the disease [9–11]. However, research on caregiver benefit finding in advanced cancer patients remains limited, and few studies have assessed caregivers during palliative care. Therefore, we interviewed caregivers of palliative care patients with advanced cancer to explore their positive experiences and benefit finding in the process of care, to improve the quality of care, and to inform intervention strategies.

## Methods

Using an exploratory qualitative design of phenomenology [18], we aim to explore the positive experiences of family caregivers of advanced cancer patients in palliative care, delving into the benefit finding they experience from it. The data were collected from February 2023 to November 2023. We adhered to the Standards for Reporting Qualitative Research checklist for our reporting [19]. The Medical Ethics Committee of Zhongnan Hospital of Wuhan University approved this study (2022127). Given the vulnerability and sensitivity of the caregivers involved in palliative care, this study strictly adheres to ethical principles to ensure the legality of the research and full respect for the rights of the caregivers. Before the interviews, the purpose, methods, and potential risks of the study were explained to each caregiver, and their informed consent was obtained. At the same time, we have promised to respect the caregivers’ right to withdraw from the study at any time. To ensure the privacy and security of the caregivers, strict measures have been taken to anonymize the data, preventing the leakage of personal information. The interview data is securely stored on encrypted devices. Additionally, our research team possesses professional counseling skills and can provide necessary support and comfort during the interviews to ensure that the caregivers’ emotions are properly cared for.

### Participants

The purposive sampling method was used to select the family caregivers of palliative care patients with advanced cancer in the oncology ward of a tertiary hospital in Wuhan as our study subjects. The inclusion criteria included being aged 18 years or older and being the primary caregiver of palliative care patients with advanced cancer during hospitalization. The caregivers were able to communicate and understand, and all participants signed the informed consent form. The exclusion criteria included the presence of psychiatric conditions, employment status, and other major stressful events that had occurred recently. The sample size was determined by the principle of data saturation, and 19 caregivers were ultimately included in this study. All participants provided informed consent and signed the informed consent form.

### Procedures

The interview guide was developed based on the research purpose and the review of existing literature and optimized through pilot interviews. The final interview outline was as follows: (a) Can you describe your experience of caregiving? (b) What difficulties did you encounter in caregiving and how did you overcome them? What support did you receive? (c) How has caregiving affected your own body, life, and family? What positive changes have occurred? (d) How has the way you relate to your patients changed? (e) What things in life are most important to you now? (f) Do you have anything else to say?

The interviews were conducted by specialty nurses who were learning and training in qualitative research methods before the interviews to ensure a standardized interview process. This study was conducted in the inpatient oncology ward, with one-on-one interviews taking place in quiet, private rooms designated for communication. Additionally, considering the convenience of the caregivers, telephone interviews were also conducted as part of the study. The purpose of the study was explained to the participants consent was obtained, and the interviews were audio-recorded throughout. The demographic information of the participants was collected, and interviews were subsequently conducted. The interviewers encouraged participants to express their thoughts and feelings deeply by listening carefully and following up with questions appropriately, and carefully observed and recorded the nonverbal messages, including tone of voice, intermittent pauses, facial, expressions, and body language. Leading or suggestive language was forbidden and any doubts in the interviews were clarified on time. Each interview lasted approximately 40 to 60 min and was adjusted accordingly based on the specific situation of the caregiver.

### Data analysis

General demographic information was collected from caregivers, utilizing frequency and composition ratios for description. Within 24 h after each interview, the audio recordings of the interviews were transcribed verbatim into text, and the information was double-checked after transcription completion. The interview transcripts were imported into Nvivo 12.0 software for coding. The Colaizzi 7-step [20] analysis method was used for data analysis. ① The researchers repeatedly and carefully read all the interview materials of caregivers, to be fully familiar with and understand what caregivers provide. ②The data are analyzed word by word, to identify and extract the relevant, important, and meaningful statements relating to the result of the benefit finding. ③ Code recurring ideas within the text, and develop general statements or explain their meanings. ④ Group similar themes and descriptions together, comparing them repeatedly to identify and extract similar ideas, thereby forming a foundational framework for the benefit finding of caregivers. ⑤ Coordinate each theme closely with the research content, extracting original statements from caregivers and providing detailed descriptions. ⑥ Compare similar themes and descriptions repeatedly, distinguishing and extracting similar viewpoints to form the basic framework of caregiver benefit finding. ⑦ Present the generated thematic structure to the caregiver for validation and feedback to ensure the accuracy of the results. Text transcription analysis and coding were independently conducted by two researchers. Subsequently, the codes were compared and cross-analysed. Any disagreements were resolved through discussion within the research group until a consensus was reached. For example, if two researchers hold different views during the coding stage or understanding of the topic, we will reach a unified understanding through discussion.

## Results

### Participants

A total of 19 caregivers participated in the interviews, one by telephone and the rest face-to-face. The average age of the patients was 54.37 (12.74) years (range: 30–82 years). The average time to cancer diagnosis was 3.58 (3.06) years (range: 1–12 years), of which 17 patients (89.5%) had metastases. The average age of the caregivers was 53.89 (14.55) years (range: 29–82 years). Most provided care for their spouses (36.8%), followed by their parents (26.3%) or children (26.3%), and a few were their sisters (10.5%). Additional details are provided in Table 1.

**Table 1:** Sociodemographic characteristics of the caregivers

Sociodemographic Characteristics - Caregiver	*N* (%)
**Gender**	**19 (100)**
Male	11 (57.9)
Female	8 (42.1)
**Educational level**	**19 (100)**
Primary or less	2 (10.5)
Junior high school	12 (63.2)
High school	3(15.8)
College and above	2 (10.5)
**Occupation**	**19 (100)**
Retired	4(21.1)
Freelancer	6 (31.6)
Farmer	4(21.1)
Employee	5(26.3)
**Living status**	**19 (100)**
Two generations together	9 (47.4)
Together with spouse	4(21.1)
Extended family	6(31.6)
**Children status**	**19 (100)**
0	2(10.5)
1	10(52.6)
2	5(26.3)
3	2(10.5)
**Comorbidities**	**19 (100)**
0	11 (57.9)
1	5(26.3)
2	3(15.8)
**Daily care duration**	**19 (100)**
9–12 h/d	3 (15.8)
13–16 h/d	3 (15.8)
17–20 h/d	3 (15.8)
21–24 h/d	10 (52.6)

## Qualitative findings

The thematic analysis revealed five main themes and fifteen subthemes reflecting benefit finding of caregivers (Table 2).

**Table 2:** Theme and subthemes

Theme	Subthemes
Personal growth	Serving as a caregiver
	Enhanced familial responsibility
	Initiating assistance
Strengthening relationships with patients	Pleasant companionship
	Increased intimacy
	Warmth and strength
Adjustment and adaptation	Energetic response
	Strengthen belief
	Patient factors
Perceived social support	Family support
	Social support
Perceived meaning in life	Cherishing companionship
	Enhance health awareness
	Maintenance of crisis consciousness
	Praying for well-being

### Theme 1: personal growth

#### Serving as a caregiver

In role change, the caregiver recognizes the importance of caregiving after the patient’s illness and takes the initiative to take on caregiving tasks and household chores.

“I treat her better than before. In the past, when we were running a pig farm and working together, I did not feel she was that vulnerable and did not pay enough attention to her. Now I do not let her do anything and just play mahjong every day. ” C12.

“She used to always take care of me and care for the family. Now that she’s sick, I take care of her as much as I can. ” C9.

#### Enhanced familial responsibility

During the caregiving process, the caregiver becomes more deeply aware of his or her family responsibilities and subsequently becomes more actively involved in family life, collaborating to maintain harmony and happiness within the household.

“I’m more mature, have a stronger sense of family responsibility, and am family-centered except for work, where mom is most important.” C14.

“I have come to understand the significance of fatherhood. While having children does not guarantee security in old age, we can still end up overwhelmed by their dependencies. Nevertheless, it is crucial to fulfill our duty towards our children, as they have been entrusted to us.” C15.

#### Initiating assistance

In the face of family difficulties, one of the caregivers expressed the selfless act of stepping up and offering help without hesitation. This selflessness is an important force in providing support.

“I will still step up to the plate when my relatives face any kind of trouble.” C7.

### Theme 2: strengthening relationships with patients

#### Pleasant companionship

The majority of caregivers reported harmonious family relationships during the caregiving process, highlighting the strong emotional connection and support they provide to their loved ones.

“I am in good health and can take good care of her. We have been getting along well over the past two years of treatment. There has always been a mutual understanding, and despite occasional friction, I can be the first to admit mistakes.” C2.

#### Increased intimacy

Caregivers spend more time with the patient, support and encourage each other, develop closer relationships, and increase the patient’s reliance on the caregiver.

“During the few years that I have taken care of her, I have done my best. She is also very understanding of me, worried that the physical demands of caring for her might wear me down. She will take care of me, remind me to pay attention to my health, and we are there for each other during hospital stays.” C6.

“She underwent surgery in 2012, radiotherapy in 2015, and targeted therapy in 2023, and she is becoming increasingly dependent on me. She wanted me to accompany her throughout the treatment. In this life, we are sisters, but in the next life, we might not be. When she came to Wuhan for treatment, I accompanied her throughout.” C7.

#### Warmth and strength

Caregivers demonstrated selfless love, valuable companionship, and personality shifts in caregiving. They all express positive coping attitudes, perceive benefits in the face of adversity, and provide mutual support to face challenges together.

“Despite the great internal pressure, the back pain, and taking care of patients at night, I still insisted on giving all my love to my daughter and treating my sick daughter wholeheartedly without any complaint.” C1.

“In the past, both my mom and dad worked outside and we usually spent little time together. However, now I have the opportunity to be with them more often, and I am especially happy to spend time with my mother.” C3.

“I used to have a bad temper, but now I always defer to her.” C12.

### Theme 3: Adjustment and adaptation

#### Energetic response

When faced with the difficult situation of a family member with a serious illness, the caregiver adjusts to the pace of the treatment, adheres to the treatment with resolute determination, has trust in the healthcare team, and maintains a positive mindset.

“She has been sick for 13 years and has grown accustomed to it. My partner always says we do not have enough money for hospital treatment. I always encourage her to keep going because the doctors have not given up yet.” C6.

Despite the pain and anxiety associated with treatment, caregivers remain hopeful, valuing the current effects of treatment and the quality of life, and are not overly worried about the uncertainty of the future.

“We are interdependent, just want to get better soon. Our mentality is very positive there is nothing to worry about. We are not facing any major challenges, nor are we overwhelmed by any burdens. ” C12.

“Before radiotherapy, I was very anxious about the patient’s condition and distressed that the patient was suffering from the disease. After radiotherapy, the treatment was effective and the pain was under control. While there is a possibility that the disease could recur, it’s something we can address when the disease can recur. There’s no need to worry about what may happen in the future.” C2.

However, some caregivers also reported that finding enjoyment in shifting their focus can help alleviate negative emotions and reduce caregiving stress.

“I often find joy in the face of adversity. Whether I’m playing cards or watching others play, I find time to go out, relax, have fun, forget my worries, and avoid crying at home.” C4.

#### Strengthen belief

Belief is a critical spiritual force that serves as the driving force and solid support for both patients and caregivers, instilling confidence in overcoming the disease.

“My daughter cannot live without my care, so this belief forces me to prioritize my health. Having cared for patients over the years, I seem to appear younger than my actual age.” C1.

“I feel like we have a great task and an enormous responsibility on our shoulders. He can only rely on us. We cannot give in we must stand by his side and fight his cancer.” C10.

#### Patient factors

The patients themselves are resilient and optimistic, enduring the pain of illness and treatment yet remaining upbeat. The caregivers profoundly influenced and sustained hope and faith, alleviating the caregiving burden.

“I accompanied her through surgery, radiation, and chemotherapy. My mom is very optimistic and strong, and I felt comfortable being there for her without much stress.” C17.

“She suffered a lot. Radiation and chemotherapy were difficult. I never heard her complain once. She was particularly resilient.” C18.

### Theme 4: perceived social support

#### Family support

Most of the caregivers reported feeling warmth during the patient’s illness with filial piety from their children and varying degrees of kindness and support expressed by relatives, friends, or neighbors.

“After she got sick, relatives, villagers, and friends showed great concern and helped us. I felt the warmth of their support. ” C9.

“When we were in financial trouble, her mother would give us some money to use.” C5.

When the caregiver is providing hospital care, other family members take the initiative to undertake household chores, care for children, parents, and other obligations, and offer encouragement and support to the caregiver.

“I suffer from hepatitis B and severe fatty liver disease, and I spend all my time taking care of my mom. Fortunately, my family is very understanding and supportive, and my exceptionally kind in-laws assist in taking care of my son and daughter. They constantly encourage me to take good care of my mom. ” C3.

#### Social support

A case where a caregiver was supported by the company by approving a caregiving leave with no loss of pay.

“Leave from work does not affect pay the unit is still very humane.” C13.

Some caregivers mentioned that insurance, health insurance, and commercial insurance provided financial support and increased the patients’ chances of treatment and prolonged survival.

“I bought commercial insurance before she got sick, and it paid out 180,000 million RMB.” C1.

“My mom has precision poverty alleviation health insurance and would not spend much on medical treatment.” C19.

### Theme 5: perceived meaning in life

#### Cherishing companionship

Faced with the uncertainty of life, caregivers cherish the present moment, do not worry too much about the future, and live with a positive attitude.

“I have to face the reality. I hope that I can live a good life and spend more time with my mother in a limited time.” C14.

#### Enhance health awareness

Caregivers can better understand the importance of good health, take the initiative to seek health-related knowledge, and pay attention to medical check-ups. The concept of seeking medical treatment should be changed, and medical treatment should be sought promptly if one feels uncomfortable. In addition, they are healthy and can take better care of patients.

“Both she and I value our health more. In the past, when I went to the hospital for examination, she would be very upset and say that I was making a mountain out of a molehill, being too worried and timid. Now she will take the initiative to ask me to have an examination.” C9.

“Taking care of my mom has made me feel that my health is so important that I will be more conscious of my physical state and focus on weight management.” C14.

#### Maintenance of crisis consciousness

Caregivers purchase commercial insurance for themselves and their family members to respond to unknown risks, avoid potential financial losses, and receive timely protection in the event of an accident.

“Enhance the sense of crisis by purchasing commercial insurance for all family members.” C19.

“At the same time, I have got commercial insurance for my dad. I hope that if there are any accidents at home, the financial piece will not be on us. ” C14.

#### Praying for well-being

Throughout the patient’s life, the caregiver is acutely aware of the patient’s significance and articulates their good wishes, hoping that the patient will recover or prolong his life, as well as wishing that the family and friends around him or her will be free from difficulties and frustrations.

“It is most important that mom is doing well on her treatment and is not resistant to medication, and I hope she can live a little longer.” C14.

“May the loved ones around me be free from hardship and frustration.” C7.

“Mom’s good health is the most important thing, but at the moment, it is no longer possible. In that case, it is crucial that she remains happy. In addition, truly, if she eats one more bite of food, I will be overjoyed.” C3.

Another caregiver hopes that a certain anticancer drug can be used in medicine as early as possible so that more people can afford it, and patients can be given more chances to treat their disease, control it, and prolong their lives.

“I hope that anticancer drugs will be included in the medical insurance plan as soon as possible.” C1.

In addition, only one caregiver reported that she did not experience any positive impact, that the patient’s personality had changed since she became ill, and that she was forceful, and assertive, and had difficulty getting along with the patient.

“She is looking at things with increasing difficulty, her mood is good one moment and bad the next, you never know when she might explode. My mom and I are very considerate of her, but she cannot seem to appreciate it. She is very dependent on me, and at the same time, she has a lot of dissatisfaction and resentment. ” C16.

## Discussion

This study explored the caregivers’ perceptions of benefit finding in palliative care patients with advanced cancer. Five themes were identified: personal growth, strengthening relationships with patients, adjustment and adaptation, perceived social support, and perceived meaning in life. The majority of caregivers reported benefit finding in the caregiving process, with only one caregiver not reporting any positive impact. This emphasizes the importance of providing psychological and social support to caregivers to help them better cope with their challenges and informs the refinement of palliative care support systems.

Cancer, as a major traumatic stressful event, has traditionally been studied mostly focusing on the negative emotions it triggers [21]. However, the Revision of Stress and Coping Theory [22] has brought positive emotions into the study of stress processes, and it reveals the coexistence of positive and negative emotions in coping with stress. Positive emotions help to restore physical, psychological, and social coping resources, and they produce positive coping processes, including seeking benefits.

The results of the study showed that caregivers experienced personal growth in caring for advanced cancer patients in palliative care, similar to the findings of Mei et al. [23]. Caregivers not only gained disease-related knowledge and skills [24] while assuming caregiving responsibilities and duties but also experienced the pleasure and satisfaction of companionship [25, 26]. In addition, by reevaluating their values taking the initiative to assist others, and embracing a role as someone who is needed, caregivers elevate their sense of self-worth and infuse their lives with deeper meaning [27]. However, caregivers may experience negative psychological changes, such as anxiety, insecurity, and helplessness, while dealing with challenges. Therefore, while promoting the growth of caregivers, it is necessary to seriously consider ways to effectively ease their burden and stress, such as enhancing the social support system, offering tailored support, and attending to their mental well-being. During this process, caregivers can achieve self-growth and cognitive improvement during the caregiving process while maintaining their physical and mental health, thus better supporting and caring for patients.

During the caregiving process, spending time together significantly strengthened the emotional bond between the caregiver and the patient, resulting in a more intimate and dependent relationship, consistent with the study by Mosher et al. [11]. Family caregivers bear a variety of physical, emotional, social, and economic burdens [28]. Enhanced relationships and enjoyable moments together foster unity, understanding, and mutual support within the family, thereby improving cohesion and well-being. Therefore, prioritizing communication and interaction among family members is crucial for enhancing cooperation, and problem-solving skills, and continually strengthening the caregiver-patient relationships.

Research has shown that caregivers’ perceptions of benefits are closely related to their positive coping styles. Consistent with the findings of Li et al. [29], positive coping mechanisms can contribute to the level of benefit finding. Optimistic caregivers are more likely to view the caregiving experience in a positive light, which ultimately leads to a greater sense of benefit [30]. Furthermore, emotional catharsis, as a form of positive coping style, can effectively alleviate stress and negative emotions. In China, the low-income population and precise poverty alleviation healthcare policies provide financial support for patients. Caregivers perceive that social support can provide them with more resources and assistance, making it easier to identify benefits and opportunities—a perspective that contrasts with the Pascoe [31] and Kangas [32] studies. However, there are still inadequacies in the social support system in our developing country. To address this, the government is actively optimizing resource allocation and enhancing policy implementation. For instance, the implementation of medical care for major illnesses and preferential policies on personal income tax can alleviate the economic burden on families resulting from medical expenses. Additionally, the government is actively promoting the development of medical alliances and strengthening collaboration between communities and medical institutions to provide caregivers with more convenient and efficient medical services. Furthermore, the study highlights the need for improved communication between patients, caregivers, and healthcare providers to ensure that caregivers have access to crucial healthcare information [33]. It is essential to enhance communication, provide relevant resources, and encourage proactive decision-making in healthcare. The acknowledgement of social support aids individuals in adopting positive and beneficial coping styles [34]. The active search for benefit finding in the face of adversity is more likely to lead to social support, which can improve one’s ability to cope with challenges and enhance adaptive capacity and psychological well-being. With the combined efforts of government, society, and individuals, we can establish a more comprehensive social support system, providing caregivers with increased support and assistance.

In the course of caregiving, caregivers gain profound insights into the fragility and resilience of life, by reevaluating its meaning, which is key to reducing their stress levels [35]. They proactively adjust their lifestyles and devise coping strategies to navigate psychological and social challenges, ultimately finding meaning and deriving benefits from it [36]. Additionally, they remain vigilant to potential crises, purchase commercial insurance, and schedule regular medical check-ups. However, in the palliative care stage, most caregivers have a vision of prolonging the patient’s life, alleviating their pain, and improving their quality of life. These experiences and understandings are helpful for caregivers to better cope with difficult situations and maintain a positive attitude.

Benefit finding has been found to be an effective strategy for coping positively with stress [37], and it is vital to maintain a positive mood, which relieves stress, enhances well-being, and improves physical and mental health [31, 38]. By strengthening the confidence of nurses and providing social support and resources, the burden of caregiving can be reduced [39]. Cognitive stress management [40], music therapy [41], group psychoeducation [42], expressive writing [43], and meaning-centered psychological interventions [44] have been proven to improve the level of benefit finding for caregivers, enabling them to gain positive influence from their caregiving experiences. These measures help reduce caregiving pressure and maintain positive attitudes among caregivers, thereby providing better support to patients.

## Study limitations

This study collected data through individual interviews, but this method has limitations. Primarily, the subjective nature of interviews can lead to respondent selection bias, which may affect the universality of research findings. Additionally, all the interviewees were from the same region and ward, which means that they might face similar circumstances and challenges, leading to similarities in their caregiving experiences. Therefore, limitations in the types of cancer diseases among the patients also limit the transferability of findings. This study uses a qualitative approach with the primary aim of gaining insight into the human experiences of a specific sample, thus the conclusions may not apply to broader populations or environments. In the future, studies should be considered in different regions and cancer types to address these limitations. It is also important to assess the extent of the caregiver’s benefit from the care using a mixed-methods research approach. This could help us explore the mechanisms behind this phenomenon and guide the development of effective interventions.

## Clinical implications

This study explored the phenomenon of benefit finding among caregivers of palliative care patients with advanced cancer, identifying five major themes that demonstrate the positive impacts that caregivers gain from their experiences with caregiving. These findings provide a valuable foundation for further research into carer benefit finding. The research findings can provide a basis for developing effective interventions targeted at caregivers to further support them in their work of caring for patients. By providing consistent support and assistance, people can enhance their coping abilities, improve their quality of life, and enable them to play a more active role in caregiving.

## Conclusions

In summary, this study highlights the benefit finding of caregivers among caregivers of palliative care patients with advanced cancer in a variety of areas, including strengthening their emotional connection with the patient, redefining their meaning of life, personal growth, and improving their coping skills. Cancer care is a long-term task, and the positive impact of caregivers—an integral part of the treatment chain—is often overlooked. Therefore, future research should pay more attention to the long-term impact of caregivers’ benefit finding.

## Data Availability

No datasets were generated or analysed during the current study.
